# Diagnostic imaging strategy for MDCT- or MRI-detected breast lesions: use of targeted sonography

**DOI:** 10.1186/1471-2342-12-13

**Published:** 2012-06-12

**Authors:** Satoko Nakano, Masahiko Ohtsuka, Akemi Mibu, Masato Karikomi, Hitomi Sakata, Masahiro Yamamoto

**Affiliations:** 1 Department of Surgery, Kawaguchi Municipal Medical Center, 180 Nishi-araijyuku, Kawaguchi-city, Saitama, 333-0833, Japan; 2 Department of Laboratory, Kawaguchi Municipal Medical Center, Kawaguchi-city, Japan; 3 Department of Radiology, Kawaguchi Municipal Medical Center, Kawaguchi-city, Japan; 4 Department of Pathology, Kawaguchi Municipal Medical Center, Kawaguchi-city, Japan

## Abstract

**Background:**

Leading-edge technology such as magnetic resonance imaging (MRI) or computed tomography (CT) often reveals mammographically and ultrasonographically occult lesions. MRI is a well-documented, effective tool to evaluate these lesions; however, the detection rate of targeted sonography varies for MRI detected lesions, and its significance is not well established in diagnostic strategy of MRI detected lesions. We assessed the utility of targeted sonography for multidetector-row CT (MDCT)- or MRI-detected lesions in practice.

**Methods:**

We retrospectively reviewed 695 patients with newly diagnosed breast cancer who were candidates for breast conserving surgery and underwent MDCT or MRI in our hospital between January 2004 and March 2011. Targeted sonography was performed in all MDCT- or MRI-detected lesions followed by imaging-guided biopsy. Patient background, histopathology features and the sizes of the lesions were compared among benign, malignant and follow-up groups.

**Results:**

Of the 695 patients, 61 lesions in 56 patients were detected by MDCT or MRI. The MDCT- or MRI-detected lesions were identified by targeted sonography in 58 out of 61 lesions (95.1%). Patients with pathological diagnoses were significantly older and more likely to be postmenopausal than the follow-up patients. Pathological diagnosis proved to be benign in 20 cases and malignant in 25. The remaining 16 lesions have been followed up.

Lesion size and shape were not significantly different among the benign, malignant and follow-up groups.

**Conclusions:**

Approximately 95% of MDCT- or MRI-detected lesions were identified by targeted sonography, and nearly half of these lesions were pathologically proven malignancies in this study. Targeted sonography is a useful modality for MDCT- or MRI-detected breast lesions.

## Background

Diagnostic procedures are crucial for the early detection of breast cancer. Advancements in imaging technology now enable us to detect mammographically and ultrasonographically occult lesions on magnetic resonance imaging (MRI) or computed tomography (CT) findings. MRI is well known for effectively detecting ductal spreading before breast-conserving surgery with excellent contrast resolution. The advantage of CT is a shorter single acquisition with extent evaluation. In Japan, CT is used for detecting the intraductal component of breast cancer as an alternative to MRI [1-4]. Multidetector-row CT (MDCT) produces imaging with a wide range and short volume acquisition time, recompounding thin slice imaging, and high-resolution and reconstructed imaging [1,2,5].

Enhancement on MDCT or MRI is due to angiogenesis and increased capillary permeability [6,7]. Enhancement of a lesion is an indication of proliferation regardless of whether it is malignant or benign. These lesions are not palpable, therefore imaging-guided biopsy is required for definitive diagnosis. There are three available ways to perform a biopsy under imaging guidance: stereo-guided, sonographically guided and MRI-guided procedures.

National Comprehensive Cancer Network (NCCN)’s guidelines have recommended the performance of MRI-guided needle sampling and/or wire localization of MRI-detected findings since 2007 [8]. Consequently, the MRI-guided biopsy is widely available in imaging centers in the United States. Although the MRI-guided biopsy is often used for MRI-detected lesions [9,10], MRI has limitations such as its low specificity, the fact that it is a time-consuming procedure, and the variation in its technical accuracy among institutions [9-12]. The availability of MRI-guided biopsy is limited in Asia, including in Japan [9-12]. On the other hand, CT-guided biopsy for MDCT detected lesions is not practical due to radiation exposure concerns [13-18].

The rationale for further modalities needed to evaluate MDCT- or MRI-detected lesions is latent formation of malignant tumors [19-25]. Since ultrasonography involves no radiation exposure, its repeated use is feasible. A second ultrasonography is performed in MDCT- or MRI-detected lesions. This use of ultrasonography is different from the initial ultrasonography involving the screening of the whole breast, and is known as targeted sonography or second-look sonography. When a lesion is detected on targeted sonography, other options in addition to MRI guidance are available, including sonographically guided biopsy, surgical excision with sonographic marking and follow-up with ultrasonography. Targeted sonography is an important tool as a breakthrough to the further examination; however, the detection rate with targeted sonography has varied in previous reports [19-24].

MDCT has been employed for routine applications in our institution since January 2004, and MRI in addition to MDCT has been employed since 2010. Since 2011, only MRI has been routinely used for the detection of ductal lesions of breast cancer due to administrative reason. In this study, we retrospectively reviewed our data regarding MDCT- or MRI-detected lesions that underwent targeted sonography followed by imaging-guided biopsy, and assessed the utility of targeted sonography in practice.

## Methods

### Patients and lesions

We retrospectively reviewed the medical records of 695 patients with newly diagnosed breast cancer who consecutively underwent MDCT or MRI for preoperative evaluation in a community hospital in Japan between January 2004 and March 2011. Mammography and ultrasonography failed but MDCT or MRI detected 31 lesions in the contralateral breast and 30 in the ipsilateral breast. An ipsilateral breast lesion is defined as a lesion that is in a different segment or more than 3 cm away from the main tumor.

We performed targeted sonography in all 61 MDCT- or MRI-detected lesions in 56 patients (8.1% in 695 patients). When targeted sonography identified MDCT- or MRI-detected lesions, we investigated cytologic or pathologic outcomes. We followed up sonographically negative lesions after consultation with the patient. Patient demographics and menopausal status were compared between the pathologically confirmed and follow-up groups. We compared the maximum tumor size and depth:width ratio of lesions detected by targeted sonography among benign, malignant and follow-up groups.

### Imaging acquisition and radiation exposure

MDCT was performed using a 16-detector row MDCT scanner (SOMATOM 16, Siemens, Germany) set for 2 mm collimation, 120 kVp, and 180 mA. Scanning was performed at 5 min and 70 seconds after the injection of contrast material (Iopamiron 370 mg/ml, Nihon Schering K.K., Osaka, Japan). Axial, coronal and sagittal images were examined, and multiplanar reconstruction (MPR) and maximum intensity projection (MIP) of the sagittal image and volume-rendering image were performed to detect the ipsilateral ductal component and breast cancer. The contralateral breast was evaluated on an axial image. The weighted CT dose indexes (CTDIw) were 7.8 mGy in plane, 14.04 mGy each in the early and delayed phases and 35.88 mGy in total.

MRI was performed on a 1.5 T scanner (Achieva, Phillips) with the use of a dedicated surface breast coil. The imaging protocol consisted of an axial fat-suppressed T2-weighted sequence in a spectral attenuated inversion-recovery sequence, an axial T1-weighted sequence, an axial diffusion-weighted sequence and an axial dynamic three-dimentional fat-suppressed T1-weighted turbo filed-echo sequence (enhanced T1 high resolution isotropic volume examination). After the injection of contrast media, a sagittal contrast-enhanced high resolution T1-weighted gradient-echo sequence was performed between 2 and 5 minutes of dynamic study. Coronal maximum intensity projection images were reconstructed from a sagital contrast-enhanced high resolution T1-weighted gradient-echo sequence.

### Breast imaging interpretation

MDCT and MRI images were independently interpreted by one radiologist with knowledge of the clinical and mammographic findings according to the BI-RADS MRI lexicon. A lesion was considered positive if there were focal and segmental enhancements, while diffuse and multiple lesions in bilateral breasts, suggesting fibrocystic changes, were considered negative.

### Sonography

Ultrasonographic examinations were performed on a LOGIQ 500 (GE Healthcare) using an 11-MHz linear transducer.

### Breast biopsy technique

We performed vacuum-assisted core needle biopsy under ultrasound guidance using 11-gauge probes (Mammotome Biopsys, Irvine, California) for definitive pathological diagnosis.

### Analysis

We used the *t*-test and chi-square test to compare mean values between two groups. For comparisons among malignant, benign and follow-up groups, we used the chi-square test and univariate analysis of variance. For multiple comparisons among the three groups, we used the Scheffé test. A value of p<0.05 was considered significant.

### Ethical considerations

This study was approved by the institutional review board of Kawaguchi Municipal Medical Center.

## Results

**Figure 1:**
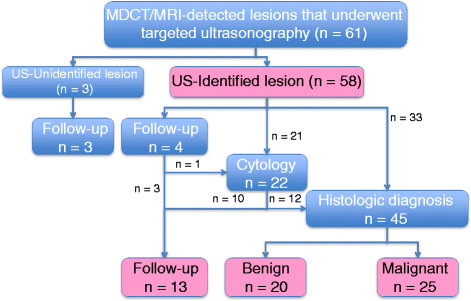
Status of Study Lesions.

Of all 61 MDCT- or MRI-detected lesions, 58 (95.1%) in 53 patients were identified by targeted sonography. Pathologic diagnoses were obtained in 45 lesions, and we followed up 13 lesions. A status of study lesions is shown in Figure 1.

As for the 53 patients, the age and menopausal status of the pathologically confirmed group and the follow-up group are shown in Table 1. Patients in the pathologically confirmed group were significantly older and more likely to be postmenopausal than those in the follow-up group. There were no significant differences in the follow-up period between the two groups (Table 1).

**Table 1:** Patient characteristics (53 patients, 58 US-identified lesions)

	**Pathologically diagnosed**	**Follow-up**	**Statistical test**
Number of patients	42	11	
Mean age (SD)	59.1 (13.2)	48.4 (13.2)	t = 2.4*
Menopausal status			
Premenopausal	14 (33)	8 (73)	Chi-square = 5.
Postmenopausal	28 (67)	3 (27)	6*

*p < .05, ns: not significant.

**Figure 2:**
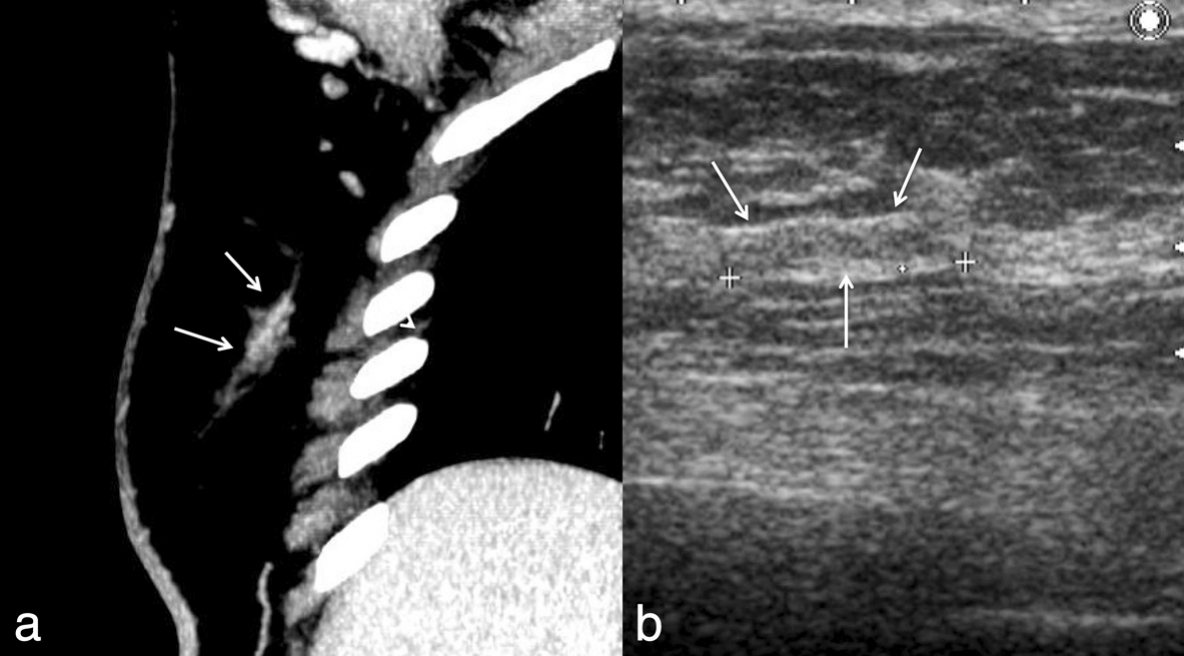
**MDCT showed segmental enhancement on the ipsilateral breast.** An enhanced lesion was depicted in the thin breast gland. Targeted sonography detected a hypoechoic lesion in the thin breast gland, the size of which was 14x2 mm. Excisional biopsy was performed for definitive diagnosis. Pathological diagnosis was ductal carcinoma in situ.

**Figure 3:**
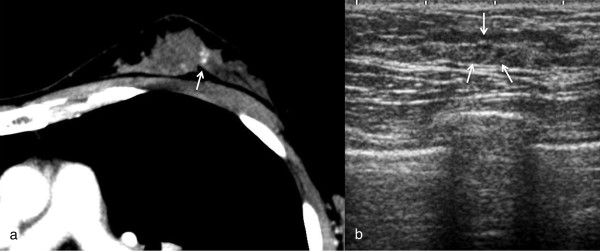
**MDCT showed clustered enhancement on the ipsilateral breast.** Targeted sonography detected a hypoechoic lesion, but it was difficult to differentiate the surrounding tissue. An excisional biopsy was performed at the same time as breast lumpectomy to obtain a definitive diagnosis. The pathological diagnosis was columnar cell hyperplasia.

**Figure 4:**
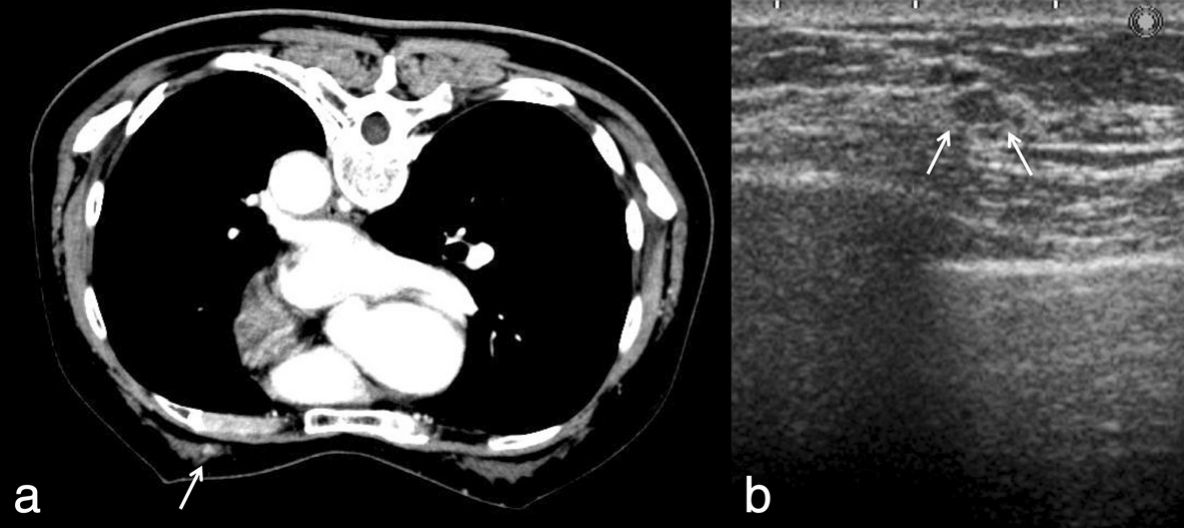
**MDCT showed enhanced focus on the contralateral breast.** Targeted sonography showed a hypoechoic mass connecting to the adjacent ducts, the size of which was 5x2 mm. Sonography-guided vacuum-assisted core needle biopsy was performed to obtain a definitive diagnosis. The pathological diagnosis was hyperplasia.

Example images of MDCT and targeted sonography are shown in Figures 2, 3 and 4. Figure 2 shows a suspicious lesion in the ipsilateral breast, and it is more than 3 cm distant from the main lesion. MDCT shows linear enhancement surrounded by fat tissues, while targeted sonography depicts a hypoechoic lesion in the atrophic thin breast with a size of 14 x 2 mm and abundant fat tissues surrounding the lesion. Ductal carcinoma in situ (DCIS) was suspected on the MDCT and ultrasonography image findings. It is often difficult to keep the lesion visible during an ultrasound-guided biopsy. We made an excision after marking on the skin of the lesion under ultrasound. The pathologic finding of this lesion was DCIS.

Figure 3 shows a clustered enhanced lesion in the ipsilateral breast on MDCT. The lesion was 1.5 cm distant from the nipple lower outer quadrant in the breast on the MDCT. Targeted sonography showed a faintly hypoechoic lesion as an enhanced area. We excised it simultaneously with performing a lumpectomy of the known breast cancer after sonographically guided marking because it was located in a difficult place to perform sonographically guided biopsy. The pathologic finding of the lesion was columnar cell hyperplasia.

Figure 4 showed a well-demarcated enhanced tumor in the thin breast tissue which was 1.5 cm distant from the nipple on the inner side. The tumor size was 5 × 2 mm. Targeted sonography depicted a hypoechoic mass in the atrophic breast and sonographically guided biopsy was performed under ultrasound guidance. The pathologic finding was hyperplasia.

Sonography-guided fine needle aspiration was performed in 22 lesions, and a pathologic diagnosis was made in 45 lesions. Of the 22 lesions, fine needle aspiration cytology was benign or normal in 7, inadequate in 7, indeterminate in 2, suspicious for malignancy in 1, and malignant in 5. For a pathological diagnosis, 30 lesions underwent sonographically guided biopsy, while 9 lesions underwent surgical biopsy during and before the operation, 5 lesions had extended excisional ranges, and one underwent core needle biopsy.

Pathologic examinations revealed benign in 20 (44%) lesions and malignant in 25 lesions (56%), including one lesion diagnosed as cancer at another hospital. Of the 20 benign tumors pathologically diagnosed in our hospital, 7 were hyperplasia, 5 were intraductal papillomatosis, 3 were fibrocystic change, 2 were fibroadenoma and one was adenoma. Proliferation was not found in 2 lesions with discharge in the dilated duct. Of 25 malignant tumors, DCIS was found in 13 and invasive ductal carcinoma in 12 (Table 2). The mean follow-up period (SD) of 56 patients was 940.2 (553.1) days.

**Table 2:** Pathologic findings in 45 lesions

	
Malignant	25
Ductal carcinoma in situ	13
Invasive ductal carcinoma	12
Benign	20
Hyperplasia	7
Intraductal papillomatosis	5
Fibrocystic changes	3
Fibroadenoma	2
Adenoma	1
No malignancy	2

The maximum diameter of the detected lesions as determined by targeted sonography is shown in Table 3. The maximum diameter was under 5.0 mm in 26 lesions, 5.1-10.0 mm in 25 lesions, 10.1-15.0 mm in 6 lesions, and over 15.1 mm in one lesion. The mean (SD) of the maximum tumor size was 6.5 (3.8) mm. As the lesion over 15.1 mm was suspected to be present in the image of one patient, we measured the size of the hypoechoic lesion. There were no significant differences in size among the malignant, benign and follow-up groups (Table 3).

**Table 3:** Maximum tumor size of the lesion detected by targeted sonography

	**Malignant**	**Benign**	**Follow-up**	**Total**	**p**
Maximum diameter (mm)					
~5.0	7 (28)	11 (55)	8 (62)	26 (45)	ns
5.1~10.0	13 (52)	8 (40)	4 (31)	25 (43)	
10.1~15.0	4 (16)	1 (5)	1 (8)	6 (10)	
15.1~	1 (4)	0 (0)	0 (0)	1 (2)	
Total	25 (100)	20 (100)	13 (100)	58 (100)	
Mean (SD)	7.7 (4.8)	5.5 (2.3)	5.4 (2.6)	6.5 (3.8)	

As shown in Table 4, the numbers of lesions with depth:width ratios under and over 0.7 were 45 and 13, respectively. There were no significant differences in the depth:width ratio in the three groups (Table 4).

**Table 4:** Depth:width ratio of the lesion detected by targeted sonography

	**Malignant**	**Benign**	**Follow-up**	**Total**	**P**
0.7≧	17 (68)	18 (90)	10 (77)	45 (78)	ns
0.7<	8 (32)	2 (10)	3 (23)	13 (22)	
Total	25 (100)	20 (100)	13 (100)	58 (100)	
Mean (SD)	.58 (.24)	.53 (.16)	.57 (.21)	.56 (.21)	

**Figure 5:**
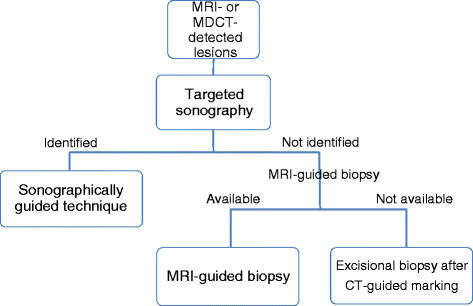
Diagnostic Imaging Strategy for MRI- or MDCT-Detected Lesions.

Based on our experience, we developed a diagnostic imaging strategy for MDCT- or MRI-detected lesions in breast cancer (Figure 5).

## Discussion

Approximately 95% of MDCT- or MRI-detected lesions were identified by targeted sonography in this study. The results suggest that targeted sonography, as a diagnostic imaging strategy, is a practical modality for all MDCT- or MRI-detected lesions, allowing more options for further evaluation. In other words, targeted sonography narrows down the number of lesions that really require MRI-guided biopsy.

Pathologic diagnosis is required for MDCT- or MRI-detected lesions because the imaging finding is not the definitive diagnosis. The MDCT- or MRI-detected lesions are not palpable; therefore biopsy is performed under imaging guidance. Regardless of the availability of MRI-guided biopsy, it is practical that targeted sonography is the first procedure to be considered for MDCT- or MRI-detected lesions. The sonographically guided technique provides an advantage. Ultrasonography is the most frequently used and easy-to-use diagnostic tool in daily practice. Once the lesion is depicted on sonography, targeted sonography provides more options for further evaluation including sonographically guided biopsy, surgical excision after marking under sonography and follow-up with ultrasonography.

The guiding principle in diagnosis depends on whether the lesion is detected with targeted sonography. Because these lesions are difficult to depict by initial mammography or ultrasonography, the detection rate of lesions with targeted sonography varies among institutions [19-24]. There are tips for detecting MDCT- or MRI-detected lesions with targeted sonography. First, the location difference due to the position should be considered. MRI is performed in the prone position, while MDCT is performed in the supine or prone position, depending on the institutions. We performed MDCT in the prone position. The positional difference is larger in the prone position than in the supine position. Attention should be paid to the deviation caused by the arm position even in the supine position. Second, the anatomical positional relation is important. The distance from the nipple, the thickness of the breast gland and the depth of the tumor in the gland should all be referenced. As the nipple is a useful milestone, we use it as a center with the medial or lateral and caudal or cranial directions used to describe locations in reference to it. Breast thickness is also helpful. These factors are not affected by positional changes. Third, tumor size, the morphology of the lesion and the structure of the breast gland surrounding the lesion are also useful information for predicting the positional difference. To determine whether the enhanced lesion is nodular, linear or segmental, and whether the breast structure surrounding the lesion is fat or abundant in the breast gland, it is helpful to perform targeted sonography. Also, these attributes are not affected by positional changes. These techniques are helpful to improve the detection rate.

There were no significant differences in the size or the depth:width ratio in the benign, malignant, and follow-up groups in our study. The mean tumor size was 6.5 mm and the mean depth:width ratio was 0.56, suggesting a relatively small and flat lesion. The MDCT or MRI detected lesion, however, has few distinct features; thus it may be found falsely negative on the initial imaging findings. We also performed cytology in 22 lesions in order to obtain a diagnosis. It is often difficult to obtain a large enough sample with fine needle aspiration, and the reliability of this technique is poor if we fail to obtain a sufficient sample. Also, cytology is not sensitive to low-grade tumors. Suspicious lesions warrant pathologic evaluation.

In this study, more than half of the pathologic findings were revealed to be malignant, and half of them were DCIS. The higher probability of malignancy requires preoperative assessment of the extent of the disease because it may alter the surgical management. We reported that MDCT contributed to the detection of occult breast cancer in 2.6% of contralateral breasts [26]. MDCT or MRI in conjunction with targeted sonography is a useful technique for detecting early carcinoma in women who are at increased breast cancer risk.

The pathologic findings revealed that there was no proliferation in 2 out of 45 lesions with discharge in the dilated duct. Because no proven proliferating lesion was found, concern remains that the lesion observed might not be the MDCT- or MRI-detected lesion. La Trenta suggests that it is not unlikely that undetected lesions are malignant [21]. It is difficult to perform core biopsy or small excisional biopsy when the lesion is not detected. At the same time, wide excisional biopsy is rather excessive treatment. A careful follow-up is always necessary to avoid delayed or missed diagnoses. The mean follow-up period for lesions unidentified by targeted sonography was 916 days in this study. So far there are no newly developed lesions or tumor growth.

It has recently been reported that real-time virtual sonography (RVS) can synchronize a sonography image and an MRI or CT image of the same section in real time [12,27]. Accurate comparison of individual positions is a useful technique for confirming a lesion. Ultrasonography greatly depends on the technique, knowledge and experience of the operator. RVS reduces the differences among operators and increases the physician’s confidence about the MDCT- or MRI-detected lesion. When the lesion is not detected with targeted sonography or RVS, MRI-guided biopsy is indicated; this option or, at the very least, follow-up is required.

We found that significantly more older patients had pathologically confirmed tumors, and they were more likely to be postmenopausal than those in the follow-up group. It is reported that younger patients have higher background parenchymal enhancement and a higher proportion of nodular parenchymal enhancement patterns [28-30]. Since younger and premenopausal groups were suspected to have fibroglandular changes, these patients require follow-up.

Our study has several limitations. It is a retrospective single-center study. Ten lesions with cytological diagnosis were not pathologically evaluated. Although fine needle aspiration cytology has high specificity and positive predictive value for mass lesions [31-33], it is difficult to obtain enough cells for MDCT- or MRI-detected lesions, particularly if it is low-grade or papillary lesion. We used fine needle aspiration as the initial diagnostic modality to provide valuable clues, but the study findings need to be interpreted with caution. We have followed-up with these patients.

## Conclusion

Targeted sonography is a useful modality for the evaluation of new unsuspected lesions found on MDCT or MRI in patients with breast cancer. By considering the previous MDCT or MRI findings and the location difference due to the position, the detection rate of targeted sonography can be improved. We achieved a 95% detection rate in this study. As a diagnostic imaging strategy, targeted sonography is a practical consideration for all MDCT- or MRI-detected lesions.

## Abbreviations

MRI, Magnetic resonance imaging; CT, Computed tomography; MDCT, Multidetector-row CT; NCCN, National comprehensive cancer network; MPR, Multiplanar reconstruction; MIP, Maximum intensity projection; CTDIw, Weighted CT dose indexes; DCIS, Ductal carcinoma in situ; RVS, Real-time virtual sonography.

## Competing interests

The author’s declared that they have no competing interests.

## Authors’ contributions

SN: has made substantial contributions to conception and design, acquisition of data, and analysis and interpretation of data; has written the manuscript. MO: has made substantial contributions to conception and design. AM: has made substantial contributions to conception and design; has performed ultrasonography. MK: has made substantial contributions to conception and design; has interpreted MDCT and MRI images. HS: has participated in the histological diagnosis. MY: has participated in the histological diagnosis. All authors read and approved the final manuscript.

## Pre-publication history

The pre-publication history for this paper can be accessed here:

http://www.biomedcentral.com/1471-2342/12/13/prepub
